# The political economy of academic publishing: On the commodification of a public good

**DOI:** 10.1371/journal.pone.0253226

**Published:** 2021-06-17

**Authors:** Stephan Puehringer, Johanna Rath, Teresa Griesebner

**Affiliations:** Institute for Comprehensive Analysis of the Economy, Johannes Kepler University of Linz, Linz, Austria; Sun Yat-sen University, CHINA

## Abstract

This paper provides an institutional and empirical analysis of the highly concentrated market of academic publishing, characterized by over proportionally high profit margins for publishing companies. The availability of latest research findings is an important issue for researchers, universities and politicians alike. Open access (OA) publication provides a promising but also costly solution to overcome this problem. However, in this paper we argue that OA publication costs are an important, but by far not the only way for academic publishers to gain access to public funding. In contrast, our study provides a comprehensive overview of the channels through which public expenditure benefits big academic publishing companies. Furthermore, we offer the results of an explorative case study, where we estimate the annual financial flows of public expenditures in Austria for the field of social sciences. In all, these expenditures add up to about 66.55 to 103.2 million € a year, which amounts to a fourth of total public funding for this field. Against this background, we contribute to the debate whether and to what extent public subsidies are justified for economically successful companies.

## Introduction

In the course of the last 25 years the movement for open access (OA) spread the claim for free access to academic knowledge [1]. While at first a rather small community of scholars supported the idea, in the last few years several initiatives for free publishing gained ground. As of today, OA has become a prominent topic in academic debates across all disciplines [2, 3] and it gets increasingly promoted by big publishing houses as well. The support as well as the reservations against OA publishing are manifold and range between two main poles: On the one hand, it is argued that OA and the rise of online-publication formats in general provide scholarly knowledge for free for all people, change academic publishing to a better and, thus, enhance scientific progress (see [4] as one pioneering example). On the other hand, the departure from traditional forms of publishing has been associated with the violation of intellectual property rights (e.g. Elseviers lawsuit against Sci-Hub see [5]), the emergence of “predatory publishers” [6] and a potential decline in the academic quality of research. However, several advancements in the field of OA publishing, such as shared definitions and conventions on OA (e.g. [7] or the Directory of open access journals [8] as well as the success of some pioneering OA outlets or archives (e.g. PlosOne; arXiv) have dispelled many reservations [9–11]. Yet, recent trends of OA are also seen ambivalent by some scholars (e.g. [12]). While on the one hand radical OA options (“OA without publishers”) could potentially challenge or even abolish power structures in science communication, these authors claim that current OA practices strictly follow the capitalist logic of commodification of academic products.

The overall success of the OA movement during the last two decades not least manifests in the vast amount of more than 14,000 and about 5 million articles listed in the DOAJ today (summer 2020). Additionally, big science funders started to mandate OA for its grantees (see 13 for the Austrian case) and new platforms for self-archiving (ResearchGate or Academic.edu) or illegal hosts (Sci-Hub and LibGen) challenge the traditional business model of academic publishers. Hence, the debate about OA is closely linked to the question of the public value and thus also the costs of scholarly knowledge as well as debates about the role of academic publishers in this field. While OA provides latest research findings for free for its readers, the costs are shifted to authors and/or their institutions. Particularly the latter–universities, libraries and consortia of both–are being confronted with increasing costs for traditional subscription fees as well as costs for OA publishing. Furthermore, the rising costs of so-called “Big-Deals” between academic publishers and distinct national consortia (e.g. the KEMÖ for the Austrian case, [13]), combining subscription and OA publication costs (see [14] for a recent study of “Big Deals” in the EU) endangers the original task of research institutions, i.e. to fulfill their procurement obligation and provide their researchers with up-to-date knowledge. The highly concentrated market of academic publishing induces and increases power differentials between academic institutions and corporate publishers to the advantage of the latter. This financial stress has also lead to some subscription cancellations of large European and U.S. universities [9, 15].

The market of academic publishing–comprising both OA and toll-access journals–overall is highly concentrated and potentially offers monopoly rents for the top publishing companies. Against this background several authors criticized the “black box” of costs for academic publishing for charging excessively, including double-dipping [16, 17] and their over proportional profit margins of about 40% [18–20]. Recently this critique has been further advanced by public media [21–23] and mainly directed towards the high level of concentration in the market of academic publishing, where only five publishing companies (Wiley-Blackwell, Springer Nature, Elsevier, ACS and Taylor&Francis—hereafter the “big five”) control up to three fourths of the market.

While most critical literature on academic publishing is focused on subscription fees, APCs and the debate on OA in general, i.e. the “revenue side” of academic publishing companies, there is hardly any literature on their respective “expenditure side”. Even critics hardly point to the fact, that academic publishers to a large extent benefit from the strong pressure to “publish or perish” [24, 25] and the inner academic practice of peer reviewing (see [19, 20] for notable exceptions). Indeed, the levels of APCs and subscription fees are very high, which has led to severe challenges for universities and libraries. Yet, these mainly publicly funded expenditures are hardly comparable to the costs of free provision of articles and peer reviews by predominantly government-funded researchers. Or in plain words: Academic publishers sell a highly profitable, yet immensely publicly subsidized product.

Against this backdrop, our study is–at least to our knowledge–the first to provide a comprehensive overview of the direct and indirect channels through which public expenditure benefits big academic publishing companies. We complement this framework with the results of an explorative case study, where we estimate the annual financial flows of public expenditures in Austria in the field of social sciences. This way, we aim to provide an empirical basis for the question, whether and to what extent public subsidies are justified for economically successful publishing companies. Moreover, we also make suggestions for a more democratic and egalitarian form of knowledge dissemination and scientific progress, alike.

The remainder of the paper is structured as follows: Section two provides an overview of the field of academic publishing and introduces our model of four channels of access to public funding for academic publishers. In section three we present some characteristics of the Austrian academic publishing market and the institutional state of the social sciences in Austria. Section four offers the main results of our case study. In section five we discuss the empirical results and provide some science policy recommendations.

## The political economy of academic publishing

The role of OA in the debate about the market structure of academic publishing is ambiguous. On the one hand OA potentially challenges the very high subscription fees of conventional academic journals. On the other hand, OA publication comes with other kinds of costs, such as individual article processing charges (APC) or general agreements (“Big Deals”) including free publications for authors. “Big Deals” between Publishing Companies and consortia provide an illustrative example of power asymmetries between buyer and seller, resulting in partly very opaque agreements: “Unfortunately, neither the countries, contracts nor data can be released due to existing non-disclosure agreements–an ironic symptom of the challenges involved in creating a transparent scholarly publishing system” [26]. Overall there are several ways of publishing OA for authors (for a comprehensive overview of different OA types see [9, 27]): Gold OA, i.e. publishing in an OA journal, where all articles are OA, hybrid OA, where the authors pay a APC to make their article publicly available, green OA, where authors are allowed to self-archive pre-prints of their article and black OA, where articles are illegally made OA (Sci-Hub being the most prominent provider). Moreover, there are many disciplinary differences as to the OA standard. While (high) APC are very common in biomedicine and other “hard sciences”, there are no APCs in the humanities and arts. The social sciences, which will be analyzed in our case study, are situated between these poles.

Despite the common argument that OA journals are forced to charge (high) APCs in order to maintain high quality standards, several studies reported only very weak or no correlation between quality of journals (measured in journal impact factors) and the level of APC. Contrarily, the level of APCs for publishing an article is more related to the market power of specific academic publishing companies and again differs strongly across disciplines [11, 18, 28]. However, the strong concentration in the market of academic publishing is similar across disciplines and varies if anything in the composition of the top publishers, depending on a stronger orientation towards natural or social science. In this regard, the Austrian Science Fund (FWF) in 2018 paid 3.82 million € for OA publications, thereof 3.33 million € for peer-review publications [29]. About two thirds of the overall amount were paid to the “big five” publishing companies. Only one pure OA publishing company—Frontiers Media–is listed in the top 8 publishers. In a similar vein, very high market concentrations have also been reported on the EU level [14, 26] as well as for different academic disciplines [16, 30]. Thus, “big five” publishers for several years not only benefit from substantial power differentials between a small number of corporate publishers and academic intuitions in the traditional academic publishing market, but are now also dominating the new evolving OA market and thus seem to have expanded their business model to this field as well [31].

### A lucrative business model for a small number of publishers

In recent decades there is a trend to understand the academic field more and more as a competitive market, mediating the scarce resource of scientific prestige. The commodification of scientific knowledge not least manifests in the denomination of the field of academic publishing as “a market”. While we share the critical stance towards this process (e.g. [12]) we use the terms “market” and “goods” because we aim to examine the financial flows in the field of academic publishing. Against the background of what was labelled an economization of science or academic capitalism [32, 33] a new form of internal stratification centered around journal impact factors and citations gained ground. Accordingly, career paths of (young) researchers are increasingly and at times exclusively determined by the simple logic of “publish or perish” [24, 25, 34]. Therefore, academic success for individual researchers as well as a good ranking position for universities is first and foremost based on a high number of publications in journals with a high impact factor. In the competitive logic of current academic capitalism tenure or better academic positions for researchers as well as the amount of government subsidies for universities strongly depend on research output as measured in impact and thus quotations. Consequently, alongside the central aim of scientific progress there is a strong incentive for researchers and universities alike, to increase their impact and prestige.

**Fig 1:**
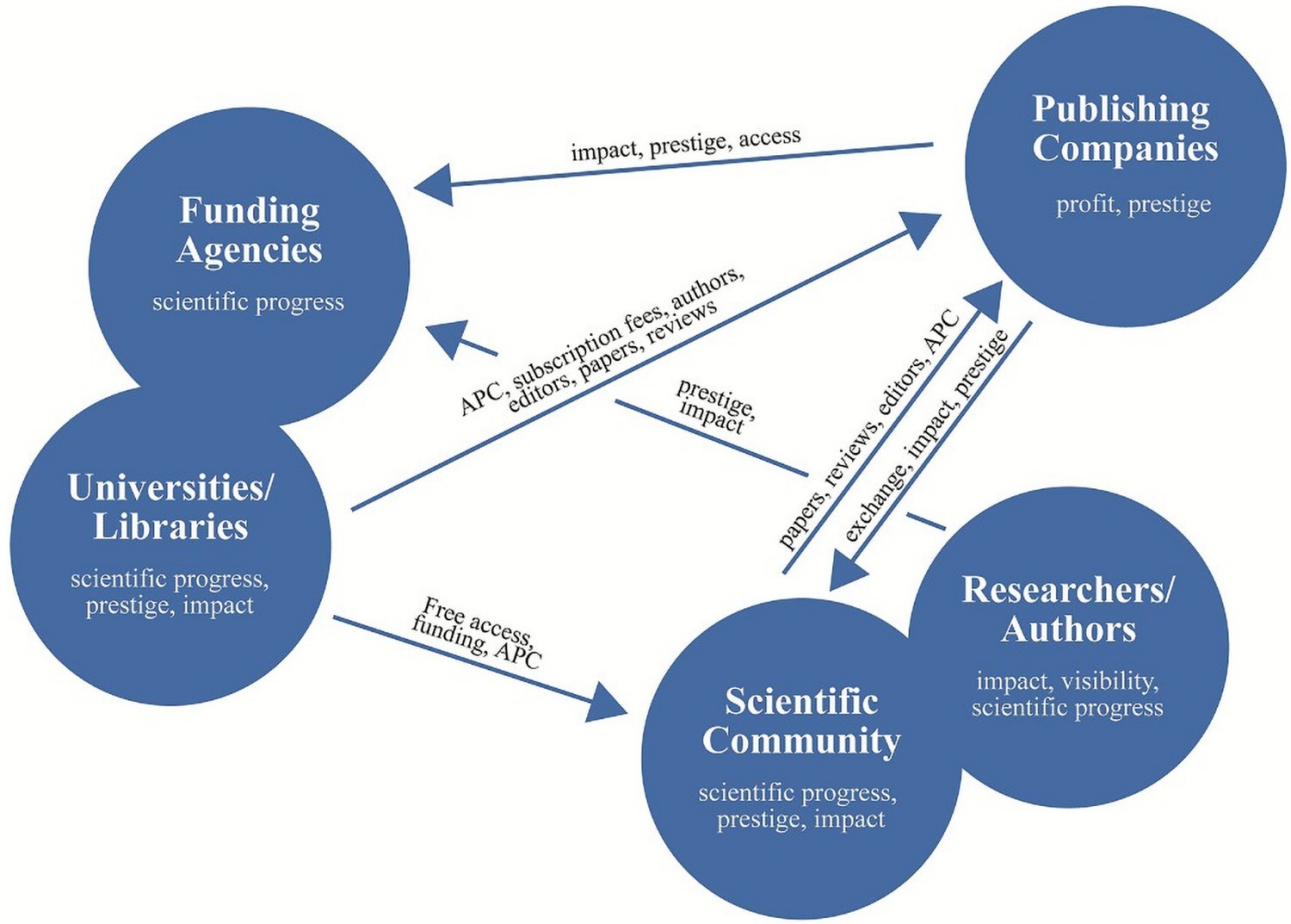
Stylized constellation of main actors in the field of academic publishing.

Throughout, one can distinguish between four different actors within the debate evolving around OA, who have partly opposing goals, claims, possibilities and perspectives: (i) authors, (ii) publishing companies, (iii) (public) funding agencies (iv) universities and libraries and (v) the scientific community. Fig 1 provides an overview of the structure of the field of academic publishing and highlights how the different actors are mutually connected to each other.

Against the backdrop of these mutual relations in the field of academic publishing, we first discuss the motivations of the main actors and then show what they offer and receive from each other. *Researchers*, as part of the *Scientific Community* of all authors, strive for visibility and impact. Therefore, *researchers* are inclined to produce papers and aim for exchange with other authors. They provide their academic impact for the prestige of their respective institution and in turn get funding, APCs and access to journals from their *universities* or *funding agencies*. Additionally, they get impact and prestige from publishing in prestigious journals, owned by *publishing companies*. Furthermore, the *scientific community* also provides reviews and editorships for academic journals and thus *publishing companies*, mainly for free. To the contrary, *universities* and *funding agencies* provide funding and free access for their researchers (and thus indirectly also provide *authors*, reviewers and editors to the *publishing companies*) and have to pay APCs, subscription fees and partly also submission fees to *publishing companies*. In turn, they receive access to scholarly publications and impact from them. Furthermore, *universities*, *funding agencies* and other non-university libraries join to national consortia and negotiate over “Big Deals” with *publishing companies* comprising free access as well as free publication opportunities for their respective authors. While the main role of *universities* and (public) *funding agencies* is to promote scientific progress, they serve a public interest and thus, particularly in Europe are mainly financed by taxes. Yet, *publishing companies* aim for profitability and prestige of their journals. They provide exchange as well as impact and prestige for publications to the *researchers* and their institutions and acquire APCs, submission fees, subscription fees but also papers, reviews and editors for free or for a very low price. Hence, *academic publishers* serve as intermediaries to enable academic exchange and ensure academic quality by mediating peer-review.

The conflictual interests of different actors in the field of academic publishing strongly influence their stance towards OA. On the one hand a shift towards OA publishing provides a promising solution to the affordability problem of *universities* and libraries and potentially reduces funding costs for public *funding agencies* and *universities*, alike. Furthermore, OA facilitates intellectual exchange among *researchers* and increases the visibility of research output–the open access citation advantage (e.g. [9, 35])—and thus increases the impact of individual *researchers*. Consequently, an increase in OA potentially promotes scientific progress and lowers costs for publicly funded *academic institutions*. On the other hand, OA has ambivalent effects for *publishing companies*. While it endangers a substantial part of the profits of *publishing companies* (subscription fees) it also offers a new business opportunity [10]: Thus, an increase in OA publishing for *publishing companies* could also induce a shift from charging readers to charging authors. However, authors of OA papers only pay for publication once, thus a paradigm shift towards OA publication could potentially challenge the very high profit margins of *publishing companies*. Although APCs have, not only within the social sciences, strongly increased during the last years, this increase could hardly compensate *publishing companies* for a stark decline in subscription revenues. Particularly, as it has been shown that the level of APC is only very weakly or not at all correlated with the quality of a journal but rather indicates the market power of specific publishers [11, 18, 28].

### A four-channels model of publisher’s access to public funding

As outlined above, the field of academic publishing reflects several trends of economization, numeric evaluations and impact rankings, which increased competitive struggles among researchers and institutions [36]. In this competitive process research output in the form of articles in high impact journals, which attract a high number of citations is the main scarce resource. Several critics have raised serious concerns about the validity of such impact rankings and their consequences for scientific investigations. These include for instance network and scale effects, incentives to publish the smallest publishable unit or other strategic behavior in the process of academic publishing ([37]; see also the recent DORA initiative to improve research assessment [38]). These developments within the field of academic publishing also produced one main side-effect: an enormous increase in academic research papers submitted to journals and accordingly a high demand for reviews to evaluate the quality of the submissions; and additionally, also an increase in the workload for journal editors. However, from a non-academic perspective rather surprisingly the great majority of these products and services is offered for free to publishing companies. In other words: mainly publicly funded researchers at universities produce research output, peer-review the quality of their products and even partly manage the reviewing process. The main motivation for the individual researcher is either simply accumulating prestige for a successful academic career or rather idealistically scientific progress as such. On top of this, publicly funded universities or funding agencies even pay again to make research output produced by themselves publicly available in order to promote scientific progress. Against this background, it hardly comes as a surprise that the profit margins of corporate publishers are obscenely high (up to 40%) in the field of academic publishing compared to other sectors [18–20].

In all, the combination of the incentive structures of the current academic system and the intrinsic motivation of individual researchers and academic institutions, offers a very lucrative business model for a small number of top academic publishing companies. In order to provide a more systematic perspective on this process, we distinguish four main channels through which publishers can receive or tap into public funding:

- **Channel 1**: subscription fees, mainly paid by university libraries
- **Channel 2**: APCs and submission fees, paid either by universities or funding agencies, in rare cases also by researchers themselves
- **Channel 3**: the provision of reviews and journal editorship free of charge
- **Channel 4**: the provision of research papers—the main input—free of charge

A similar study was conducted by Lawson et al., who examine UK publishing markets including three broad types of financial flows (institutional income, subscription payments and APCs) between the different actors involved in scientific publishing markets [16]. These financial flows correspond to channel 1 and 2 in our schematic “channels-model”. The 2-sided market power results from the specific structure of scientific publishing markets. Publishers profit from the oligopolistic structures on the market of subscription and submission fees (see e.g. [30]). In this setting, few offering (high) impact journals meet a large number of demanding subscribers, which results in market power to charge mark-ups and thus high subscription (channel 1) and submission fees (channel 2). On the other end, on their supply market, few publishers face a high number of offering authors and reviewers, who depend on the publisher’s service of publishing. Their supply for papers and reviews, the publisher’s final goods and quality control, is highly inelastic as the number of products offered does not seem to react to a change in payments but is rather based on intrinsic motivation of researchers and reviewers to support the scientific community. Therefore, publishers hold an oligopolistic market position on their supply side and a monopsonist position on their demand side, giving them a strong standing of market power due to the market structures of scientific publishing.

Against the background of our analysis of power differentials and financial flows in the field of academic publishing, we employ our schematic “channels-model” for an explorative case study of public funding of research output in the field of social sciences in Austria. Hence, we contribute to the debate on the role of public funding in academic publishing and furthermore provide an original estimation of the actual annual financial flows in this particular market.

### Academic publishing in the social sciences in Austria

While much of the critical debate on academic publishing, i.e. the critique against the increase of APCs and subscription fees initially focused on the natural sciences, the social sciences were confronted with rising costs more recently as well. In a study conducted in 2013 the authors found that about 18% of social science journals enlisted in the DOAJ charge APCs, whereas this share is 80% in genetics [39]. Regarding the power differentials among academic publishing companies the “big five” also have a dominant position within the field of social sciences. More specifically, we found that about two thirds of the top journals in the respective SSCI categories are owned by five publishing companies. However, unsurprisingly ACS with its sole focus on natural sciences is replaced by SAGE. In what follows we provide an explanatory case study of the financial flows in academic publishing for the field of social sciences in Austria. Against this background, we first provide some stylized facts of the current state of social sciences in Austria.

The social sciences are composed of several disciplines dealing with human behavior in its social and cultural aspects. However, this broad definition aggravates the assignment of distinct sub-disciplines and researchers to the field. In order to arrive at a clear definition of social scientists in Austria, in this survey we use the classification of Social Science by the Austrian Science Fund FWF, which is based on international standards and includes the fields psychology, economics, pedagogy, sociology, legal studies, political science, human geography, media and communication studies and other social studies (see the Appendix for a list of institutes included in our survey).

We decided to include only researchers with a PhD and thus ended up with a full sample about 1,500 social scientists in Austria. In sum, accounting for 427 out of 2617 professorships at 22 public universities in Austria, the social sciences constitute about one sixth of the Austrian research sector [40]. This proportion is also reflected in public expenses: In 2017, 2.5 billion € were invested in research and development at universities across all scientific fields. Out of these, 382.9 million € (15%) went to social sciences, of which 94% were funded by the public sector. This means that in 2017, 359.1 million € were invested in research and development in social sciences by the public sector [41].

Big deals with publishing companies in Austria are negotiated by the KEMÖ (Austrian Academic Library Consortium). Currently 58 Austrian libraries are part of the consortium and their contracts include 61 publishers. Four of the big five publishers in general and social sciences (ACS, Wiley, Springer Nature, Taylor&Francis and SAGE) have contracts with the KEMÖ that include open access agreements. In general, the deals have a term of three years. The precise amount of money negotiated is confidential [42].

**Fig 2:**
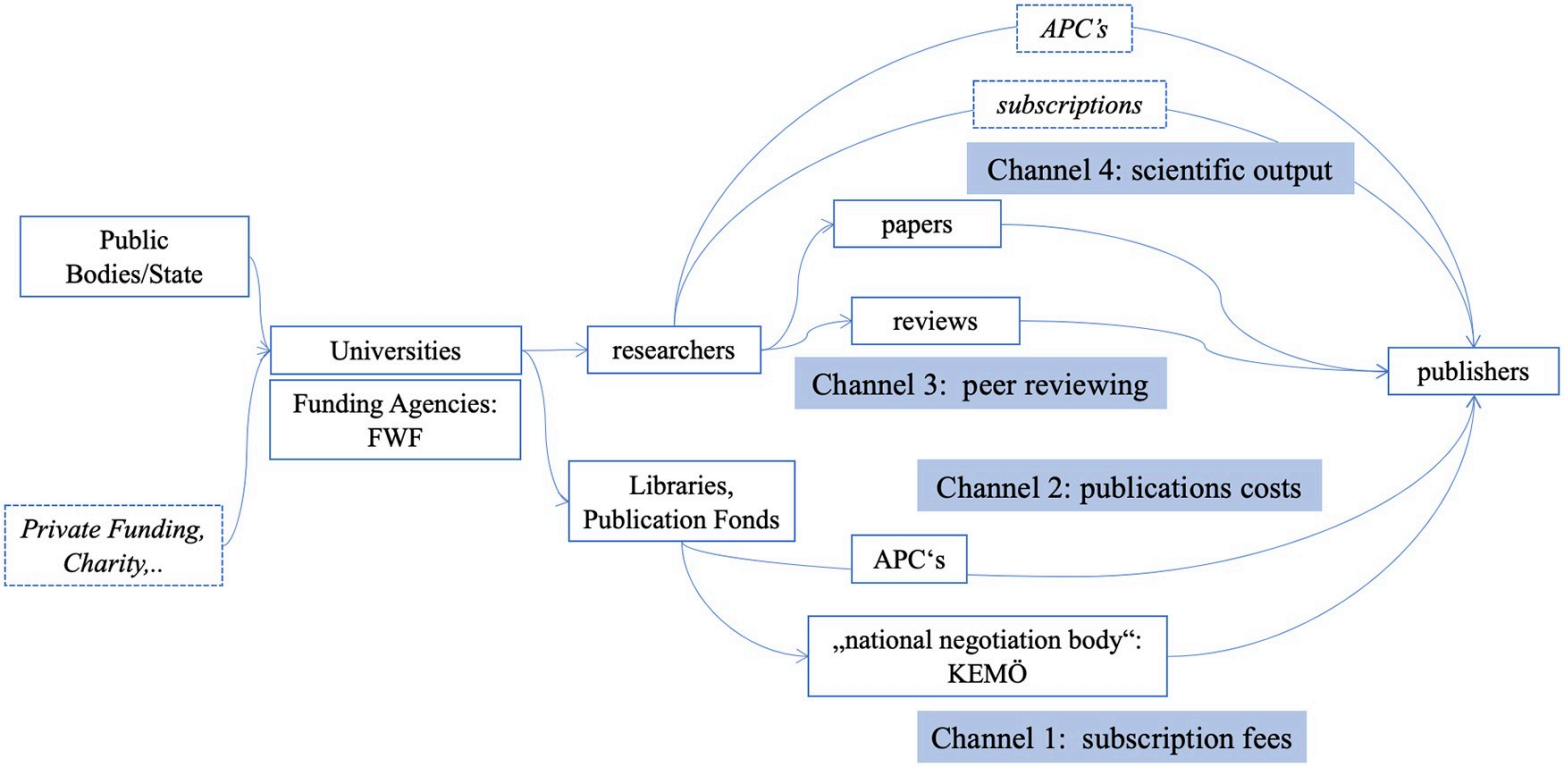
Four channels of access to public funding for academic publishers. This representation is based on [18].

The aim of this study is to highlight structures of financial flows in scientific publishing for the field of social sciences in Austria. Based on the four-channels-model (see Fig 2) presented earlier the following section deals with estimates about the actual amounts of money flowing through these channels. The goal is to come up with an aggregated number of estimates of financial flows from public institutions and funding bodies to private publishers, including (1) subscription fees, (2) APCs and submission fees, (3) the value of peer reviewing as well as (4) the value of scientific papers.

## Research design and methodology

The study rests on two methodological pillars: First, we examined several previous studies concerned with the topic of publishing costs in a systematic framework and categorized them in accordance with our four-channels-model. Secondly, we gathered primary data from a questionnaire study conducted among a full sample of Austria social scientists to supplement our analysis. We arrive at an estimation of public expenditures related to payments according to the four-channels-model by combining those two sources of information. The presented results heavily depend on the given institutional and cultural context of Austrian scientific publishing in social sciences. We therefore do not claim for representativeness or universality of our results. Still, the case study contributes to the overall discussion by illustrating an empirical example of theoretical considerations about power concentration and oligopolistic structures on scientific publishing markets [30, 43].

### Meta-analysis of cost structures in the field of academic publishing

We started by screening the existing literature on scientific publishing and public expenditures related to publishing. Several studies have dealt with the topic, although from slightly different angles and based on different data sets and time periods. In order to achieve better comparability, we limited our analysis to articles which calculate empirical estimates of expenditures of scientific publishing, excluding for example theoretical discussions of market structures. We ended up including 22 articles into the analysis and coded them with regard to statements made about the amount of subscription fees and libraries expenditures (channel 1), APCs and publication fees (channel 2), costs of reviewing (channel 3), and costs/article (channel 4). Most of the studies included in the analysis derive descriptive statistics and estimators of direct costs associated with scientific publishing, that is subscription fees (channel 1) and submission fees (channel 2). Whenever several countries or disciplines were discussed in the original paper, we focused on numbers derived for Austria and the field of social sciences. By combining several different studies and their results we hope to gain a broader and more realistic picture on the annual amounts spent on subscription and submission fees, as well as on the extent of divergence of these numbers between studies using different approaches and data sets. Additionally, we performed an online search for subscription and submission fees published on the journals websites to present the most recent official figures (Table 5).

Several constraints have to be taken into account when comparing the numbers from the literature depicted in Table 1. First, most of the studies are based on different scopes as well as multiple data bases. In order to counteract this variety of sources and features, we transformed some of the figures using information from the original papers in order to achieve better comparability, while all other values are directly taken from the original papers. Furthermore, many studies used aggregated data (e.g. EUA) for quantitative statistical analysis. Eventually, most studies mentioned a lack of data or other problems of identification that often stem from incompleteness in the data due to disclosure clauses concerning big deals about subscription and submission fees between publishers and academic libraries or public negotiation bodies like the KEMÖ in Austria.

**Table 1:** Studies on costs and expenditures in the field of academic publishing.

*studies*	*Channel 1*		*Channel 2*		*Channel 3*	*Channel 4*
	Subscription costs	Libraries expenditure	Publication fees	Average APC	costs of reviewing	cost/article
[44]		€ 39,8447– € 225,8116 (*)				€ 0.14–0.68 /page
[45]				€ 1,684.02–3,586	€ 1,242.45–1,391.54	€ 7,857.81–10,265.40
[46]		72% of publishers revenues		€ 822,40–2,4672	e. g. € 82.24 (paid to reviewers)	€ 0.12/ page
[47]			€ 252.47	€ 685.63		
[11]			€ 1,644.80–3,2896	€ 743.45 (€ 202.31–1,1061)		€ 904.48 (*)
[48]		average annual price increase: 13%	€ 8.2 billion/ year (€ 575 Mio in SoSci)	e.g. € 2,467.20 (Springer)	€ 500 (hypothetical cost estimate)	
[19]			€ 1,110.24 /paper (PlosOne);	€ 485.65 (for OA publishers)		e.g. € 238.50 (Hindawi), € 24,6720–32,916 (Nature)
[49]	€ 1 Mio/ university /year					
[50]	€ 65–70 Mio/year	€ 30 Mio (KEMÖ)				
[13]	€ 30 Mio/year (universities); € 1.5 Mio/year (author’s pay)		€ 3.9 Mio /year (*)			
[10]	€ 218 Mio/year—260 Mio/year		€ 92 Mio—144 Mio	€ 1,100		
[51]	€ 30 Mio/year	€ 60–70 Mio. /year	€ 0.9Mio—1.5 Mio. / year	€ 3.5 Mio / year		
[30]		68–75% of journal publishing revenues from library deals		€ 4,1145 (Cell Reports by Elsevier)		€ 16.46–32.92 /page
[52]	e.g. € 527.09 (economic journal); € 604.83 (AEA)	€ 4.9 Mio / year / library on journal subscriptions; e.g. € 9.8 Mio for Elsevier’s collection		€ 1,110.92 (PLoS ONE); € 1,657.50 (Economic Journal);		e.g. € 1,234.35 (Wiley) € 8.229–24,687 (Nature) € 1,110.92 - € 2,386.41(PloS)
[53]				€ 1,6574– € 2,7624		
[2]	€ 418,408 (total costs of full OAfunding)	€ 2,376,356 (funding by FWF)	€ 1,000–1,250	€ 1,298		€ 4,816.18 (*)
[16]	€ 103.8 Mio/year (UK)	€ 19 billion (*)		€ 1,878.50		
[54]	€ 2,037.63 (*)		€1,481.22–2,304.12			
[18]				€ 2,240.76–2,634.10		€ 660.55
[14, 26]	€ 597 Mio (EU), € 451 Mio (Big5)					€ 1,526
[55]				€ 2,147.01–2,653.11		
[56]					€ 41 Mio (€ 28.79/ review)	

In order to achieve better comparability, we converted all costs into Euro at the first published exchange rate in the year of the study. Converted numbers are marked with a (*). For example, if the number of articles and the total volume of revenues were given in the original paper, we calculated the corresponding amount of revenues/article or the mean value of different business model’s amount of fees for this table.

Table 1 presents the main findings of our meta-study of relevant literature. A detailed discussion of the results is presented in the following section.

### A survey among Austrian social scientists

Since the vast majority of articles in the existing literature deals with channels 1 and 2, we decided to collect additional data on channels 3 and 4 in order to estimate implicit public expenditures related to reviewing and producing manuscripts, the main “goods” traded on academic publishing “markets”. Therefore, we collected primary data by designing a questionnaire and forwarding it to all social scientists (see the Appendix for a full list of institutes) with Austrian affiliations starting from PhD-Level. We collected the first round between 4^th^ of February 2020 and 15^th^ of March 2020 and a second round between 17^th^ of August and 7^th^ of September 2020. The link to the online anonymized questionnaire was connected to a serial number in order to exclude multiple participations per scientist. External lectors or social scientists working at Universities of applied sciences (*Fachhochschulen*) or comparable Austrian research institutes were excluded from the sample. Further, we clustered them into a total of five positions, including project staff, Post-Docs, Assistant Professors, Associate Professors and University Professors.

In sum, we identified 1496 social scientists affiliated to Austrian universities and invited them to join our survey. The gross response rate was 15.7%, the net response rate 10.56%. Demographics of the participating sample are given in Table 2.

**Table 2:** Demographics.

GENDER						
**male**	**female**		**divers**	**not reported**		
90	51		1	16		
POSITION						
**Associate Professor**	**Assistant Professor**	**University Professor**	**Post-Doc**	**Project Staff**	**others**	**unspecified**
21	25	44	49	1	5	13
DISCIPLINE						
**Sociology**	**Statistics**	**Business Administration**	**Economics**	**Political Studies**	**Business Informatics**	**others**
21	4	35	39	13	4	46

The main focus of the questionnaire was the amount of time spent on writing a paper, i.e. time spent on producing the publisher’s final good, as well as the amount of time spent on reviewing, i.e. time spent on the quality control of the scientific output of others. This approach aims to capture the proportion of scientific activity in terms of working time that is actually connected with the production of a paper (or a review respectively).

**Fig 3:**
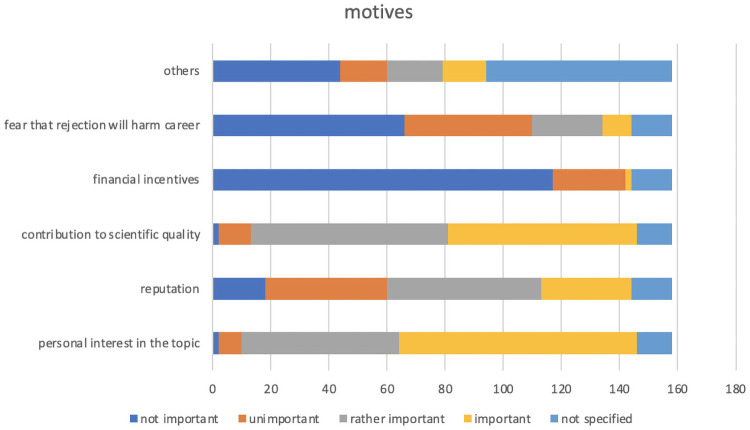
Intrinsic motivation for reviewing. The bars indicate the number of individuals rating the different motives according to their personal importance.

An additional check on the individual’s motivation provides deeper qualitative insights on the incentive structure of individual researchers. When asking if and how important several motivational reasons are to actually perform a review, about 90% (89.87%) stated that financial incentives are unimportant or not important for their decision. Further, fear of potential harm on their personal careers if rejecting to write a review was also rather unimportant or unimportant to a majority of researchers (69.62%). On the contrary, contributing to the quality of science and personal interest in the topic were rated very important and rather important by about 85% of researchers in the sample. These qualitative insights to the intrinsic motivation of reviewers show that market incentives and mechanisms might be largely ineffective in this case, as the supply of reviews is practically independent from the price or compensation paid. Even if compensation is zero, scientists still review for the sake of the scientific quality process and out of personal interest. Other motives often stated in the survey and summarized in Fig 3 under the heading “others” can be categorized in reasons of collegiality, time restrictions, commitment to the journal (such as e.g. being a member of the journals Advisory Board), Ethics and Fairness (e.g. reciprocity), the perception that reviewing is “part of the game” and personal positive effects (such as networking or learning effects from reviewing).

Several authors have tried to come up with an estimate of unit costs of an article for scientific publishing from the publisher’s perspective that is the cost side of publishing. However, such derivations evoke several problems, e.g. due to different structures and sizes of the individual publishers (for a list of arguments against unit cost calculation of articles see [57]). For this reason, we chose to tackle this problem of data availability from a different side, that is we asked scientists how much of their working time they spent on producing publisher’s final goods (papers and reviews). As wages for scientists at Austrian universities, and thus the main element of the costs of production and quality control of a scientific article, are almost exclusively financed by public funds, this approach better suits our research question (Table 3).

**Table 3:** Descriptive statistics for some selected key figures from the case study.

	share of scientific work/day	articles published (2019)	hours/ review	numbers of reviews written (2019) for publishers:		requests for reviews / year
				conventional	OA	
**mean**	46%	3.37	7.35	6.5	1.05	12.43
**median**	42%	3	6	4.5	0	7
**min**	7%	0	1	0	0	0
**max**	91%	25	50	40	10	100

For comparison, in 2017 the Austrian Union of University Professors (UPV) published a study on the amount of time spent on actual research activity by university professors [58]. Their results suggest that only 25% of the total working time is spent on research activities (including project application). In our sample the average share of scientific work on an average day was indicated with 46%. This figure reduces to 35% in our sample if only university professors are considered and is therefore slightly higher than the numbers elevated by the UPV study. However, we consciously chose a wide interpretation of the term “scientific activity” to grasp the broad spectrum of activities that contribute to the creation of a single scientific paper. These include activities within the process from theory to proposing a hypothesis to the verification of the hypothesis but indirectly also efforts put in previous research with negative and thus often unpublished result.

In order to come up with an estimate of indirect public expenditures on scientific output passed on to publishers, we used monthly gross wages for full time employment according to the collective agreement for public employees and adjusted them for the average numbers of hours employed in our sample and the average share of working time spent on researching and reviewing. The average employment indicated in the sample was 38.09 hours/week. Furthermore, the average wage per hour was calculated combining the 4 different wage models’ (Post-Doc, Associate Professor, Assistant Professor, Professor) minimum wage as derived from the collective agreement for public employees according to the distribution of the 4 positions among Austrian based social scientists (see Table 4 for the single calculation steps). Dividing the calculated minimum (maximum) wage costs/month of € 6,904.68 (€ 10,272.75) by the average employment of 38.09 hours weekly, gives a minimum average hourly wage cost for social scientists in Austria of € 41.38 and a maximum average hourly wage for social scientists in Austria of € 62.23 (Table 4).

**Table 4:** Wage costs of social scientists.

Position [Class]	number	Share of researchers	min. wage costs [€]	max. wage costs [€]
**Prof [A1]**	427	28.54%	9,303.24	13,345.44
**Assistant [A2]**	112	7.49%	6,787.88	8,029.62
**Associate[A3]**	244	16.31%	8,786.44	12,841.24
**Post-Doc [B1]**	713	47.66%	4,842.62	7,905.96
**sum**	1496	100.00%		
		**Weighted wage share**	**6,904.68 €**	**€ 10,272.75**

The monthly wages were derived from the website of the union of public employees [59]. We use them to calculate the total wage costs for the universities, i.e. gross wages plus incidental wage costs. The variation is caused by the length of the employment contract.

## Discussion of results

In this section we will proceed as follows: For the estimation of financial funds related to channel 1 and 2 we make use of the available information from our literature survey on publication and subscription costs. Channel 3 and 4 are estimated by combining these results with data from our case study. Hence, applying our four-channels model we come up with a rough estimate of the actual amount of money flowing from public institutions to private publishing companies.

### Channel 1: Subscription costs

As mentioned above, one considerable restriction in analysing subscription fees is that academic institutions, such as university libraries, often do not pay the list price due to internal deals that cover a great variety of journal subscriptions, often also including extra deals on free submission to some of their journals. Therefore, there is an almost universal demand for more transparency regarding true institutional costs of subscription fees in the literature dealing with open access and scientific publishing. We also tried to come up with actual numbers and contacted the KEMÖ several times, but did not succeed in getting additional information. Further, the range of numbers in Table 1 presented above gives an idea about the great variety in business models among different journals and publishers. The spectrum of sales models ranges from online-only subscription packages, to print-only and any possible combinations of those, as well as special prices for students, scientists with affiliation in the Global South, and many more.

[54] estimated average individual subscription fees of € 423,16 for non-profit (economic) journals to € 3,652.10 for for-profit journals. However, subscriptions to journals on an individual level are a rather marginal phenomenon, as most subscriptions are organized in Big Deals with libraries and consortia. For example, institutional subscription revenues account for 72% of journals total subscription revenues in 2007. Among those, 5 top journals account for 28% of total subscription revenues [46]. The actual numbers of these Big Deals are not publicly available; however, several studies calculated estimates of institutional subscription costs and library expenditures for subscription fees.

Overall institutional expenditure on subscription were calculated by [49], who come up with an estimate of average annual payments of € 1 million/university/year, which correspond to a 40% discount of the € 2.5 million according to list prices for subscription. [13] investigate subscription costs especially focusing on Austria, and come up with annual expenditures of € 30 million for university subscription and an additional € 1.5 million for individuals (author’s pay). Similarly, [50] reported annual subscription costs of € 30 million paid by KEMÖ in Austria and estimated total institutional subscription costs to € 65–70 million. This comes close to [51] with a more conservative estimate of € 48 million total institutional expenditures, thereof € 30 million on subscriptions only. [16] calculated a total of € 103.8 Mio/year for institutional subscriptions in the UK, and [14] report a total of € 597 million/year for subscriptions within the EU, thereof € 451 million to the big five publishers. The estimated total annual expenditures on institutional subscription per country thus vary from approximately € 30 to 70 million in the literature. Since the share of social sciences in Austrian academia is approximately one sixth, the estimated amount of subscription fees expenditures in the field of social science in Austria ranges from € 5 to 11.7 million/year.

*Channel 1*: The estimated of amount of annual subscription fee expenditures in social sciences in Austria vary between € 5 million and € 12 million.

### Channel 2: Publication costs

The term submission fees (similarly publication costs) typically summarize various forms of payment for submitting to a journal, that is submission fees, Article Processing Charges (APCs), Open Access fees and others. A broad definition is given by any form of payment made by the author (the author’s institution respectively) to make the article available to the scientific community–this implies both, paying publication fees to a journal, or paying additional fees to make the article OA. APCs in the top journals in the field of Social Sciences range from € 2,495 to € 5,350 in 2020 according to the official list prices published on the publisher’s website, as presented in Table 5. As argued above, the quality of papers and journals cannot sufficiently explain the striking differences in prices (see e.g. [18]). Alternatively, the concentrated market structures and the resulting market power of individual publishers to set prices might be reasons for the price differentiations.

**Table 5:** Publication costs for top social science journals.

Rank	Journal	Publisher	submission fees	APC
**1**	Academy of Management Annals	academy of management	0	No gold OA
**2**	Annual Review of Organizational Psychology and Organizational Behavior	Annual Reviews	n/a	n/a
**3**	Entrepren€ship Theory and Practice	SAGE	-	€ 2,450
**4**	Journal of International Business Studies	Palgrave	-	n/a
**5**	Journal of Management	SAGE	0	€ 2,450
**1**	Annual Review of Sociology	Annual Reviews		n/a
**2**	American Sociological Review	SAGE	€ 20.42	€ 2,450
**3**	Annals of Tourism Research	Elsevier	-	€ 2,352
**4**	Information Communication & Society	Taylor and Francis	0	€ 2,495
**5**	Sociological Methods & Research	SAGE	-	€ 2,450
**1**	Political Communication	Taylor and Francis	0	€ 2,495
**2**	International Organization	Cambridge University Press	-	no OA
**3**	Environmental Politics	Taylor and Francis	0	€ 2,840
**4**	American Journals of Political Science	Wiley	-	€ 2,750
**5**	Political Analysis	Cambridge University Press	-	€ 2,581.40
**1**	Quarterly Journal of Economics	Oxford Academy Press		€ 5,350
**2**	Journal of Economic Perspectives	American Economic Association		
**3**	Economic Geography	Taylor and Francis		€ 2,495
**4**	Brookings Papers on Economic Activity	Brookings Institution Press		
**5**	Journal of Finance	Wiley	€ 0–245	€ 2,962

Manually compiled list of top 5 of Web of Science ranked Journals in Social Science categories according to their Journal Impact Factor [60], online research. Converted into Euro.

The findings of literature study on publications costs provides further insights into the structure and amount of submission fees. Some differences in the level of APCs can be attributed to different modes of access: For example [45], differentiate between Open Access/Non-OA and print/online. They find that OA charges the lowest average APC € (1,827.43) followed by online-only mode (€ 2,802.30) and print&online mode (€ 3,893.48). Another factor determining the APC level is the institutional background of publishers: [11] distinguish on the basis of publisher’s profit orientation and find the lowest average APC in universities journals (€ 189.41) followed by scientific journals (€ 354.94) and commercial publishers (€ 1,035.57).

Several studies have derived overall estimates for average per article APCs. The lowest estimate is given by [19] with € 485.65, followed by [47] estimating an average APC for all OA publishers in their sample of € 685.63. [10] estimate average APC of € 1,100, [2] state € 1,298, [16] calculate € 1,878.50 for UK universities [54], derive an APC of € 1,481.22–2,304.12 for gold OA fees and [18] state the highest average APC in our meta-survey of literature that is € 2,240.76–2,634.10/article. Overall, estimates of average per article APC in Table 1 range from € 485.65 to € 2,634.10/article. In fact, most of the differences are caused by different disciplinary conventions, different modes of access (e.g. gold/hybrid, online/online&print, etc) or different institutional backgrounds and profit-orientation of the publishers.

Additionally, several authors came up with estimates of total costs of institutional expenditures on submission fees and publication costs. For example [10], derive a hypothetical total amount of annual payment of € 140 million in Germany, € 144 million in the UK and € 92 million in France. [13] focus on expenditures by the FWF and state expenditures on hybrid OA journals of € 2.4 million/year and € 1.5 million/year on gold OA, summing to a total of € 3.9 million on publication fees. This is consistent with an estimate by [51] of € 3.5 million expenditures for open access by the FWF, and an additional € 0.9–1.5 million for other publication fees, summing to a maximum of € 5 million of possible expenditures on submission fees by the FWF. Additional expenditures for books with an amount of approximately € 3–4 million, are estimated overall publication costs (including APCs) of € 7.5–9 million [51]. [48] come up with a total volume of the global publishing market for Social Sciences of € 575 million/year.

Downscaling the estimate of [51] for Austria of € 7.5–9 million to the field of Social Sciences by 1/6 gives total institutional expenditures on submission fees ranging from 1.25 million to 1.5 million € /year.

*Channel 2*: The annual amount of submission fees in Austria among the social sciences is between € 1.25 million and € 1.5 million.

### Channel 3: Peer reviews

Although much research has been done in the area of subscription and submission fees, the role of the reviewing process has been little studied to date. [45] analyse various publications costs and identify costs of proof-reading, reviewing and other editing tasks sum to € 1,242.45–€ 1,391.54/article published. Further [56], analysed the peer reviewing process and conclude a total of € 41 million costs of peer reviewing, € 28.79/ review. They also gather data on the hours spent on a review, summing to 5 h / review.

In our sample, 94.94% of scientists indicated to have already produced a review in the course of their career. On average, approximately 16.7 inquiries for writing a review have been received in 2019 by a single scientist and 6.5 reviews for commercial publishers as well as 1.05 reviews for OA publishers have actually been provided in 2019, summing to approximately 7.55 reviews/scientist/year. The estimated time for one review was 7.35 hours on average, which is close to the estimates in the literature.

To calculate the value of reviews provided by Austrian social scientists in one year we multiplied the average hourly wage of min € 41.83 (and max € 62.23 respectively) with the average number of hours spent/review (7.35 h). This results in an approximate value of a single review of € 168 to € 239. Multiplying this with 7.55 reviews written (6.5 for commercial as well as 1.05 for an open access publisher on average in 2019) gives a public expenditure for reviewing of € 2,323 to € 3,456 / year / social scientist in Austria. This again multiplied with the total number of social scientists in Austria (1496) sums up to an annual total value of reviewing by Austrian social scientists of € 3,299,366 to € 4,908,779.

*Channel 3*: The amount of (indirect) expenditures for reviewing in Austria in the field of Social Sciences in 2019 ranges between € 3.3 million and € 4.9 million.

### Channel 4: Scientific output

As mentioned above, only few estimates for unit costs of articles can be found in the literature, most of them focusing on the production costs for publishers to edit and publish an article. This way, most of these studies focus on average profit margins in scientific publishing markets and thus aim to identify costs and revenues of publishers. For example [30], state costs of € 16.46 to 32.92 /page. In [19] several experts are interviewed, for example people employed at Hindawi (one of the largest Open Access Publishers), estimating costs of publishing/article to € 238.50, or employees of Nature Journal stating cost estimates of € 24,6720- to € 32,916. He argues, that these high discrepancies are due to the very different rejection rates of the two journals, as a higher rejection rate implies higher costs/published article. Other studies dealing with costs associated to publishing are [18] reporting € 660.55 /article and [14] estimating € 1,526/article, however the latter two do not take the costs of rejected papers into account. However, as we are interested in the value of scientific output offered to publishing companies and thus the (indirect) public costs of these publications, we estimate the value of an article written in a similar way as estimating the value of reviews above. Thus, we estimate the value of an article by calculating the effort that is put into research resulting in a scientific output.

On average, our respondents indicated a share of research activity that aims at producing a scientific output (journal paper, chapter, book or other manuscripts) on a normal working day by 46%. Combined with the average weekly employment of 38.09 hours, the total number of hours spent on research activity by an average social scientist in Austria is approximately 911 hours/year, producing 3.37 contributions on average in 2019. One weakness of this approach is that non-published, or even unfinished papers are not considered, nor corrected for and that therefore the numbers presented might be overestimated for the numbers of hours spent on producing published articles. Nevertheless, we argue that research on unpublished work might indirectly benefit the published work.

The approximate value of a scientific contribution produced by Austrian social scientists is calculated with a minimum and maximum wage (see Appendix for the exact calculation). The resulting range of the value of a scientific contribution by an Austrian social scientist spans from € 38,114 to € 56,706. The resulting range of the value of scientific activity in a year for all social scientists in Austria spans from € 57,018,318 to € 84,831,539.

*Channel 4*: The amount of (indirect) expenditures for scientific contributions in Austria in the field of social sciences in 2019 ranges between 57 million € and 85 million €.

## Conclusion

The contribution of this paper is twofold: First our paper provides a comprehensive overview of the direct and indirect channels through which public expenditure benefits big academic publishing companies. In doing so, we build a four-channel-model of publisher’s access to public funding and thus contribute to the political economy of academic publishing. Second, we use this model for an empirical case study of the financial flows in field of social sciences in Austria. While the open access movement initiated an ongoing debate and several positive developments regarding channel 1 and 2 of our model–subscription fees, APCs and submission fees–channel 3 and 4, i.e. the free provision of peer reviews and research papers is largely understudied. Therefore, we supplement the analysis of the “revenue side” of publishing companies (channels 1 and 2) with their “cost side” (channels 3 + 4), which allows us to develop a broader understanding of the political economy of academic publishing.

More specifically, we focus on the role of academic publishing companies. We argue that a very small number of these companies benefit from the strong stratification logic, the “publish or perish” culture in academia and the academic practice of peer reviewing. During the last years, publishing companies have steadily increased the levels of APCs and subscription fees, which has led to severe challenges for universities and libraries. Moreover, recent studies have shown that these “big five” companies make use of the quasi-oligopolistic position to force libraries, universities and consortia to pay increasing fees in order to maintain their main role as provisionary of academic knowledge.

Against this background, our four-channels model identifies the implications of this huge power differentials between publishers and researchers as well as academic institutions on the one hand and several problematic incentive structures in academia on the other hand. Based on previous studies in the field of academic publishing and a questionnaire study of a full sample of Austrian social scientists, we estimate the annual public expenditures reaped by publishing companies. This way, we are able to estimate the amount of public funding which is explicitly (channel 1 and 2) or implicitly (channel 3 and 4) directed to a few dominant publishing companies.

**Fig 4:**
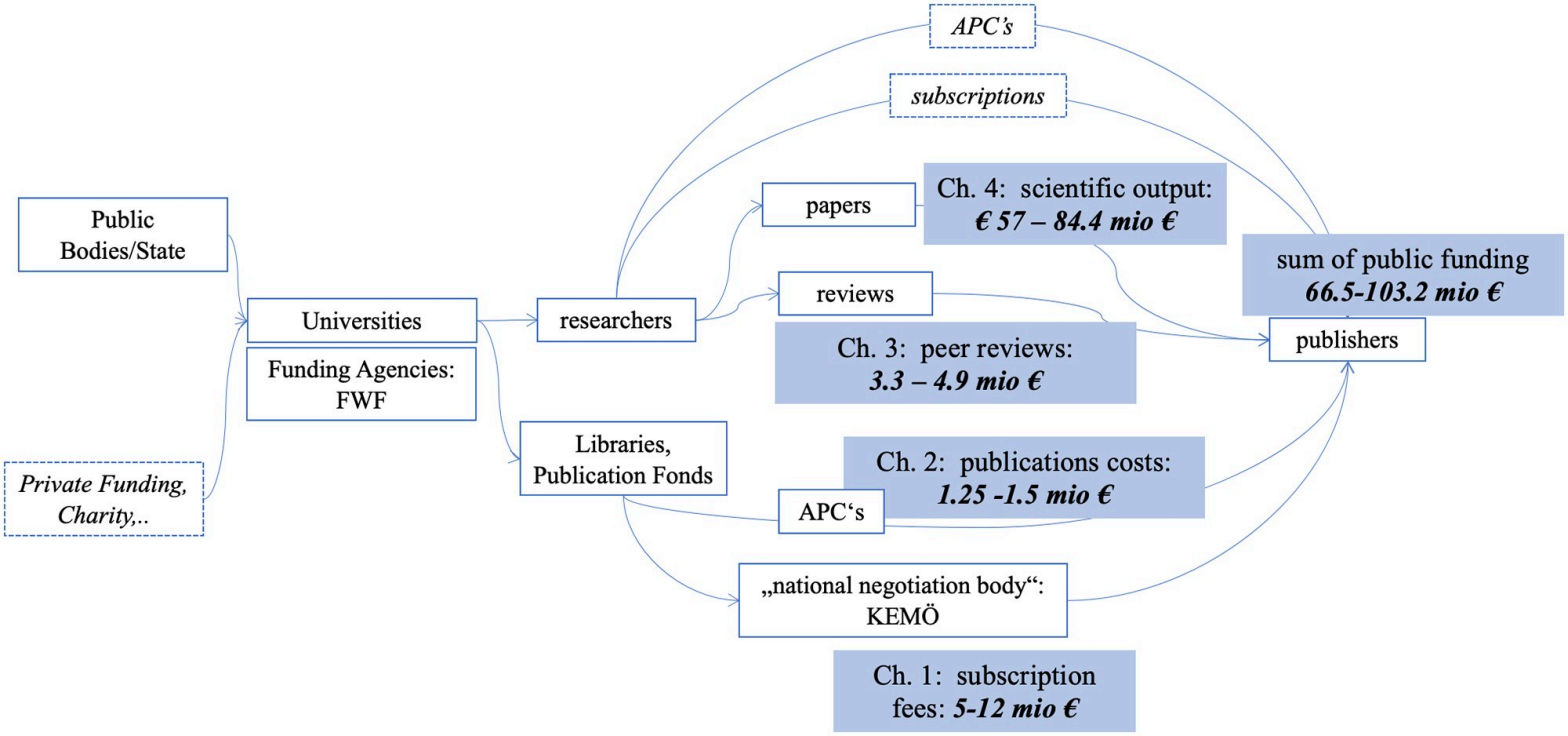
Four-channel-model of access to public funding for academic publishers. An estimation for the social sciences in Austria.

Summing up the estimations for our 4-channels model, our estimation suggests that the Austrian state (indirectly) funds publishing companies in the field of Social Science with € 66.55 to € 103.2 million per year (Fig 4). This amount corresponds to about a fourth of the annual basic funding Austrian universities receive from the Ministry of education, science and research in the field of social sciences.

Although our results have to be interpreted with caution–not least due to the lack of transparency in the agreements between publishing companies, universities and consortia–our case study provides some novel insights into the political economy of academic publishing. We argue that in the highly concentrated market of academic publishing a small number of publishing companies (“big five”) benefit from the highly competitive academic culture and the intrinsic motivation of individual researchers.

Against the background of our empirical results we conclude that academic publishing is in urgent need of institutional reform. Apparently, despite the success of the open access movement in recent years, academic publishing is still a very lucrative business for a very small number of private academic publishers. Yet there are already some initiatives to challenge the problematic implications of the huge power differentials in the market of academic publishing and the problematic incentives for academic publishing, alike. While increasing obligations to publish open access could challenge the traditional business model of academic publishers, initiatives for a better and more comprehensive evaluation of research output [38] are a promising road to tackle the destructive academic culture of publish or perish.

## Supporting information

S1 Appendix Questionnaire. (DOCX)

S2 Appendix Calculation of value of scientific activity in social sciences in Austria. (DOCX)

S3 Appendix Social science sample. (DOCX)

S4 Appendix Minimal data set. (XLSX)
