# Supplementary material for: The political economy of academic publishing: On the commodification of a public good
Source: PLoS One. 2021 Jun 17;16(6):e0253226. doi: 10.1371/journal.pone.0253226 (PMC8211248; doi:10.1371/journal.pone.0253226)
Supplement: S1 Appendix — (DOCX) [file pone.0253226.s001.docx]

**S1 Appendix. Fragebogen/Questionnaire.**

| **Part 1: Introduction** | | |
| --- | --- | --- |
|  | **German (original language)** | **English** |
|  | Einleitung, allgemeine Informationen zur Studie, Datenschutzerklärung | Introduction, general information on the survey, Data Protection Declaration |
| **Part 2: Questions and outcomes** | | |
| **Question 1** | „Bitte geben Sie das Stundenausmaß Ihrer Anstellung an.“ | „Please indicate the number of hours of your employment.“ |
| **Outcome 1**  **(open)** | __ Stunden/Woche | __ hours/week |
| **Question 2** | „Welchen Anteil an Ihrer gesamten Arbeitszeit machen Forschungstätigkeiten an einem durchschnittlichen Arbeitstag tatsächlich aus?  Geben Sie den Anteil an tatsächlich verrichteter Forschungstätigkeit (d.h. Literatursuche, Lektüre, Verfassen wissenschaftlicher Arbeiten, Datenauswertung, etc) an einem durchschnittlichen Tag an.“ | „On average, what share of your total working time does research activity actually account for in a working day?  Please indicate the proportion of research activity (i.e. searching for literature, reading and writing scientific papers, data analysis, etc.) carried out on an average day.“ |
| **Outcome 2**  **(open)** | __ % Anteil der Forschungstätigkeit/Tag | __ % share of research activity/day |
| **Question 3** | „Wie viel Zeit verbringen Sie mit der Lektüre von wissenschaftlichen Beiträgen (in Fachjournalen, Sammelbänden, etc) an einem durchschnittlichen Arbeitstag?  Geben Sie die Anzahl der Stunden an, die Sie für das Lesen von Fachliteratur im Durschnitt pro Tag aufwenden.“ | „How much time do you spend reading scientific contributions (in journals, books, etc.) on an average working day?  Please indicate the average number of hours you spend on scientific literature per day.“ |

| **Outcome 3**  **(open)** | __ Stunden/Tag | __ hours/day |
| --- | --- | --- |
| **Question 4** | „Wie viele wissenschaftliche Beiträge haben Sie im letzten Kalenderjahr/insgesamt in Fachjournalen, Sammelbänden, etc (mit Reviewverfahren) veröffentlicht?  Geben Sie jeweils die Anzahl der von Ihnen veröffentlichten Beiträge in Fachjournalen, Sammelbänden, etc im letzten Kalenderjahr an, sowie insgesamt im Laufe Ihrer Karriere an.“ | „How many scientific contributions have you published in scientific journals, books, etc. (reviewed) in the last calendar year and in total?  Please indicate the number of papers and contributions you have published in scientific journals, book, etc in the last calendar year and in total during your career.“ |
| **Outcome 4**  **(open)** | __ veröffentlichte Beiträge im letzten Jahr  __ Beiträge insgesamt | __ papers published in the last year  __ papers published in total |
| **Question 5** | „Beiträge in internationalen peer-reviewten Fachjournalen werden in den Wirtschafts- und Sozialwissenschaften immer zentraler. Haben Sie bereits ein Gutachten im Rahmen eines Peer Review Verfahrens zu einem Beitrag aus einem Fachjournal, Sammelband oder Monographie etc aus Ihrem Forschungsfeld erstellt?“ | „Papers in international peer-reviewed journals are becoming increasingly important in economics and social sciences. Have you already written a peer review on a paper from a scientific journal or book etc. in your field of research?“ |
| **Outcome 5**  **(single choice)** |  ja   nein   keine Angabe |  yes   no   not specified |
| **Question 6** | „Wie viele Anfragen für die Erstellung eines Gutachtens im Rahmen eines Peer Review Verfahrens bekommen Sie durchschnittlich in einem Jahr?  Geben Sie eine Schätzung über die an Sie gerichteten Anfragen für die Erstellung eines Gutachtens im Rahmen eines Peer Review Verfahrens pro Jahr an (unabhängig davon, ob die Anfrage angenommen wurde oder nicht).“ | „How many requests to write a peer review do you receive on average in one year?  Provide an estimate of the number of requests addressed to you for writing a peer review per year (regardless of whether or not the requests were accepted).“ |
| **Outcome 6**  **(open)** | ___ Anfragen/Jahr | ___ requests/year |
| **Question 7** | „Sind Sie als EditorIn in einem Fachjournal in Ihrem Forschungsbereich tätig?“ | „Are you an editor in a scientific journal in your research area? “ |
| **Outcome 7**  **(single choice)** |  ja   nein   keine Angabe |  yes   no   not specified |
| **Question 8** | „Welche Motive waren für Sie ausschlaggebend ein Gutachten zu erstellen/nicht zu erstellen? Bewerten Sie die Bedeutung der genannten Motive bei Ihrer persönlichen Entscheidung für oder gegen die Erstellung eines Gutachtens.”  **Motive:**   - persönliches Interesse am Themengebiet - Ansehen/Reputation - Beitrag zur Qualität wissenschaftlicher Forschung - finanzielle Anreize - Befürchtung, dass eine Ablehnung der wissenschaftlichen Karriere schadet - weitere Motive: ____ | „What motives have been decisive for you to write / not to write a review? Evaluate the meaning of the motives mentioned in your personal decision for or against writing a peer review.“  **motives:**   - personal interest in the topic - prestige / reputation - to contribute to the quality of scientific research - financial incentives - concern that rejection might harm the academic career - additional motives: ___ |
| **Outcome 8**  **(single choice)** |  nicht wichtig   eher unwichtig   eher wichtig   sehr wichtig |  not important   rather unimportant   rather important   very important |
| **Question 9** | „Haben Sie im letzten Kalenderjahr individuell Zahlungen für den Zugang zu Online-Journalen (bzw Online-Artikeln) getätigt?“ | „Have you made individual payments to get access to online journals (or online papers) in the past calendar year?“ |
| **Outcome (single choice)** |  ja   nein   keine Angabe |  yes   no   not specified |
| **Question 10** | „Besteht an Ihrer Forschungseinrichtung die Möglichkeit zur Förderung von Publikationen über einen Publikationsfonds (o. Ä.)? Möglichkeiten zur Förderung von Publikationen schließen alle Formen finanzieller Unterstützung beim Publizieren von Fachartikeln durch die Universität und/oder den/die DrittmittelgeberIn ein.“ | „Is your research facility able to fund publications through a publication fund (or similar)? Possibilities for funding publications include all forms of financial support for the publication of papers by the university and / or third-party funding.“ |

| **Outcome 10**  **(single choice)** |  ja   nein   keine Angabe |  yes   no   not specified |
| --- | --- | --- |
| **Question 11** | „Wurden im letzten Kalenderjahr Zahlungen für Publikationen von Ihnen getätigt? Bitte wählen Sie die Arten der getätigten Zahlungen, Mehrfachnennungen sind möglich“ | „Have you made payments for publications in the calender yearpast? Please indicate the form of payments made. Multiple answers are possible.“ |
| **Outcome 11**  **(multiple choice)** |  ja, Open-Access-Gebühren __ €   ja, Publikationsgebühren __ €   ja, Einreichgebühren __ €   nein |  yes, open access fees __ €   yes, publication fees __ €   yes, submission fees __ €   no |
| **Part 3: General information** | | |
| **Demographics** | Alter, Position, Geschlecht | Age, Position, Gender |
| **Scientific experience** | Jahr der Promotion, Disziplin | Year of PhD, Discipline |
|  | Anmerkungen | comments |
