# Supplementary material for: The political economy of academic publishing: On the commodification of a public good
Source: PLoS One. 2021 Jun 17;16(6):e0253226. doi: 10.1371/journal.pone.0253226 (PMC8211248; doi:10.1371/journal.pone.0253226)
Supplement: S2 Appendix — (DOCX) [file pone.0253226.s002.docx]

**S2 Appendix. Calculation of value of scientific activity in Social Sciences in Austria.**

**Calculation of value of scientific activity in Social Sciences in Austria**

| Months/year | 12 |
| --- | --- |
| Weeks/year | 52 |
| Hours/week | 38.09 |
| **Hours/month** | **165.07** |
| **Minimum monthly wage** | **€ 6,904.68** |
| **Minimum average hourly wage** | **€ 41.83** |
| **Maximum monthly wage** | **€ 10,272.75** |
| **Maximum average hourly wage** | **€ 62.23** |

**Calculation of annual value of reviewing in Social Sciences in Austria**

| **Reviewing:** | **Min wage** | **Max wage** |
| --- | --- | --- |
| Wage/hour: | € 42.83 | € 62.23 |
| Wage/hour*hours spent on reviewing: | € 307.50 | € 457.50 |
| Value of review / reviewer: | € 2,323.08 | € 3,456.27 |
| Value of all reviews by social scientists 2019: | € 3,299,366.14 | € 4,908,778.71 |

**Calculation of annual value of writing scientific papers, books, etc**

| **Scientific Papers:** |  |  |
| --- | --- | --- |
| annual hours spent on research: | 911.19 |  |
| min wage: | 42.83 |  |
| max wage: | 62.23 |  |
|  | **min** | **max** |
| value of research activity/year/scientist: | € 38,113.85 | € 56,705.57 |
| value of research activity/year: | € 57,018,318.14 | € 84,831,538.63 |
