# Supplementary material for: The political economy of academic publishing: On the commodification of a public good
Source: PLoS One. 2021 Jun 17;16(6):e0253226. doi: 10.1371/journal.pone.0253226 (PMC8211248; doi:10.1371/journal.pone.0253226)
Supplement: S3 Appendix — (DOCX) [file pone.0253226.s003.docx]

**S3 Appendix. Social science sample.**

**List of Institutes and Faculties included in the Social Science sample, 2019**

| **University of Graz** |
| --- |
| **Sozial- und Wirtschaftswissenschaften:** |
| Institut für Banken und Finanzierung |
| Institut für Finanzwirtschaft |
| Institut für Informationswissenschaft und Wirtschaftsinformatik |
| Institut für Marketing |
| Institut für Organisation und Institutionenökonomik |
| Institut für Personalpolitik |
| Institut für Produktion und Logistik |
| Institut für Soziologie |
| Institut für Statistik und Operations Research |
| Institut für Unternehmensführung und Entrepreneurship |
| Institut für Unternehmensrechnung und Controlling |
| Institut für Unternehmensrechnung und Reporting |
| Institut für Unternehmensrechnung und Steuerlehre |
| Institut für Unternehmensrechnung und Wirtschaftsprüfung |
| Institut für Wirtschafts-, Sozial- und Unternehmensgeschichte |
| Institut für Wirtschaftspädagogik |
| BANDAS-Center |
| Center for Social Research |
| Fachbereich Volkswirtschaftslehre |
| **Umwelt, Regional- und Bildungswissenschaften:** |
| Institut für Erziehungs- und Bildungswissenschaft |
| Bildungsforschung und PädagogInnenbildung |
| Wegener Center für Klima und Globalen Wandel |
|  |
| **University of Klagenfurt** |
| **Fakultät für Wirtschaftswissenschaften:** |
| Institut für Finanzmanagement |
| Institut für Innovationsmanagement und Unternehmensgründung |
| Institut für öffentliche BWL |
| Institut für Organisation, Personal und Dienstleistungsmanagement |
| Institut für Produktions-, Energie- und Umweltmanagement |
| Institut für Soziologie |
| Institut für Unternehmensführung |
| **Fakultät für Kulturwissenschaft:** |
| Institut für Erziehungswissenschaft und Bildungsforschung |
| Institut für Medien- und Kommunikationswissenschaft |
| Institut für Psychologie |
|  |
| **University of Linz** |
| **Sozial- und Wirtschaftswissenschaftliche Fakultät:** |
| Institut für betriebliche Finanzwirtschaft |
| Institut für betriebliche und regionale Umweltwirtschaft |
| Institut für Betriebswirtschaftliche Steuerlehre |
| Institut für Controlling & Consulting |
| Institut für Digital Business |
| Institut für Handel, Absatz und Marketing |
| Institut für Innovationsmanagement |
| Institut für Internationales Management |
| Institut für Management Accounting |
| Institut für Organisation |
| Institut für Personalführung und Veränderungsmanagement |
| Institut für Produktions- und Logistikmanagement |
| Institut für Public und Nonprofit Management |
| Institut für Strategisches Management |
| Institut für Unternehmensgründung und Unternehmensentwicklung |
| Institut für Unternehmensrechnung und Wirtschaftsprüfung |
| Institut für Wirtschaftsinformatik/Communications Engineering |
| Institut für Wirtschaftsinformatik/Data & Knowledge Engineering |
| Institut für Wirtschaftsinformatik/Information Engineering |
| Institut für Wirtschaftsinformatik - Software Engineering |
| Institut für Volkswirtschaftslehre |
| Institut für die Gesamtanalyse der Wirtschaft |
| Institut für Angewandte Statistik |
| Institut für Gesellschafts- und Sozialpolitik |
| Institut für Sozial- und Wirtschaftsgeschichte |
| Institut für Soziologie |
| Institut für Pädagogik und Psychologie |
| **Cross-Faculty:** |
| Institut für Frauen- und Geschlechterforschung |
|  |
| **University of Innsbruck** |
| **Fakultät für Betriebswirtschaft:** |
| Institut für Rechnungswesen, Steuerlehre und Wirtschaftsprüfung |
| Institut für Banken und Finanzen |
| Institut für Wirtschaftsinformatik, Produktionswirtschaft und Logistik |
| Institut für Organisation und Lernen |
| Institut für Strategisches Management, Marketing und Tourismus |
| **Fakultät für Volkswirtschaft und Statistik:** |
| Institut für Wirtschaftstheorie, Wirtschaftspolitik und Wirtschaftsgeschichte |
| Institut für Finanzwissenschaft |
| Institut für Statistik |
| **Fakultät für Bildungswissenschaft:** |
| Institut für Erziehungswissenschaft |
| Institut für Psychosoziale Intervention und Kommunikationsforschung |
| **Fakultät für Psychologie und Sportwissenschaft:** |
| Institut für Psychologie |
| **Fakultät für Soziale und Politische Wissenschaften:** |
| Institut für Medien, Gesellschaft und Kommunikation |
| Institut für Politikwissenschaft |
| Institut für Soziologie |
|  |
| **Vienna University of Economics and Business** |
| **Drittmittel:** |
| Institut für Altersökonomie |
| Institut Economics of Inequality |
| **Department für Volkswirtschaftslehre:** |
| Instituts für Analytische VWL |
| Institut für Arbeitsmarkttheorie und -politik |
| Institut für Außenwirtschaft und Entwicklung |
| Institut für Finanzwissenschaft und Öffentliche Wirtschaft |
| Institut für Makroökonomie |
| Institut für Institutionelle und Heterodoxe Ökonomie |
| Institut für Internationale Wirtschaft |
| Institut für Quantitative Volkswirtschaftslehre |
| Institut für Volkswirtschaftspolitik und Industrieökonomik |
| **Department für Sozioökonomie:** |
| Institute for Multi-Level Governance and Development |
| Institut für Sozialpolitik |
| Institute for Ecological Economics |
| Gesellschaftswandel und Nachhaltigkeit |
| Institut für Nachhaltigkeitsmanagement |
| Recht und Governance |
| Soziologie und Empirische Sozialforschung |
| Wirtschaftsgeographie und Geoinformatik |
| Wirtschafts- und Sozialgeschichte |
| **Department for Finance, Accounting and Statistics:** |
| Institute for Accounting & Auditing |
| Institute for Finance, Banking and Insurance |
| Financial Research |
| **Department für Informationsverarbeitung und Prozessmanagement:** |
| Information and Management Control |
| Institute for Information Business |
| Produktionsmanagement |
| Wirtschaftsinformatik und Gesellschaft |
| Wirtschaftsinformatik und Neue Medien |
| **Department für Management:** |
| Change Management and Management Development |
| Gender und Diversität in Organisationen |
| Nonprofit Management |
| Organization Studies |
| Personalmanagement |
| Public Management und Governance |
| Interdisziplinäres Institut für Verhaltenswissenschaftlich Orientiertes Management |
| Wirtschaftspädagogik |
| **Department für Marketing:** |
| Handel und Marketing |
| Interactive Marketing & Social Media |
| Internationales Marketing Management |
| Marketing Management |
| Service Marketing and Tourism |
| Institute for Marketing and Consumer Research |
| **Department für Strategy and Innovation:** |
| Institute for Corporate Governance |
| Institut für Hochschulmanagement |
| Institut für Entrepreneurship & Innovation |
| Markets and Strategy |
| Organization Design |
| Strategie, Technologie und Organisation |
| Strategisches Management |
| Unternehmensführung |
| Abteilung für Projektmanagement |
| **Department für Welthandel:** |
| International Business |
| KMU Management |
| Transportwirtschaft und Logistik |
| **Kompetenzzentren:** |
| Emerging Markets |
| Empirische Forschungsmethoden |
| Gründungszentrum |
| STaR Sustainability Transformation and Responsibility |
| Nonprofit-Organisationen und Social Entrepreneurship |
|  |
| **University of Vienna** |
| **Fakultät für Wirtschaftswissenschaften:** |
| Department of Decisions and Analytics |
| Institut für Finanzwirtschaft |
| Institut für Marketing und International Business |
| Institut für Rechnungswesen, Innovation & strat. Management |
| Institut für Recht der Wirtschaft |
| Statistik & Operations Research |
| Institut für Wirtschaftssoziologie |
| Vienna Center for Experimental Economics |
| Vienna Center for Operations Research |
| Institut für Volkswirtschaftslehre |
| **Historisch-Kulturwissenschaftliche Fakultät:** |
| Institut für Wirtschafts- und Sozialgeschichte |
| **Fakultät für Philosophie und Bildungswissenschaft:** |
| Institut für Bildungswissenschaft |
| **Fakultät für Psychologie:** |
| Institut der Psychologie für Kognition, Emotion und Methoden |
| Institut für Arbeits-, Wirtschafts- und Sozialpsychologie |
| Institut für Psychologie der Entwicklung und Bildung |
| **Fakultät für Sozialwissenschaften:** |
| Institut für Demografie |
| Institut für Internationale Entwicklung |
| Institut für Kultur- und Sozialanthropologie |
| Institut für Pflegewissenschaft |
| Institut für Politikwissenschaft |
| Institut für Publizistik und Kommunikationswissenschaft |
| Institut für Soziologie |
| Institut für Staatswissenschaft |
| Institut für Wissenschafts- und Technikforschung |
|  |
| **University of Salzburg** |
| **Kultur- und geisteswissenschaftliche Fakultät:** |
| Fachbereich Erziehungswissenschaft |
| Fachbereich Kommunikationswissenschaft |
| Fachbereich Politikwissenschaft und Soziologie |
| Center of European Union Studies |
| **Rechtswissenschaftliche Fakultät:** |
| Bereich Strategisches Management und Organisation |
| Bereich Marketing und Innovation |
| Bereich Human Resource Management |
| Bereich Finanzmanagement und Finanzdienstleistungen |
| Bereich Rechnungslegung und Steuerlehre |
| Bereich Volkswirtschaftslehre |
| **Naturwissenschaftliche Fakultät:** |
| Fachbereich Psychologie |
|  |
| **University of Krems** |
| **Fakultät für Wirtschaft und Globalisierung:** |
| Department für Wirtschafts- und Managementwissenschaften |
| Department für E-Gonvernance in Wirtschaft und Verwaltung |
| Department für Migration und Globalisierung |
| Department für Wissens- und Kommunikationsmanagement |
| Department für Europapolitik und Demokratieforschung |
| **Fakultät für Bildung, Kunst und Architektur:** |
| Department für Weiterbildungsforschung und Bildungstechnologien |
